# Exploring the Mediating Role of Working Alliance in a Peer Support Intervention for Late-Life Depression

**DOI:** 10.21203/rs.3.rs-9139788/v1

**Published:** 2026-04-02

**Authors:** Jin hui Joo, Samuel Van Vleet, Fei Wu, Phyllis Solomon, Nicholas Cloney, Alice Xie, Joseph Locascio, Namkee Choi, Ryan Mace

**Affiliations:** Massachusetts General Hospital; Massachusetts General Hospital; Harvard University; University of Pennsylvania; Brigham and Women's Hospital; Massachusetts General Hospital; Massachusetts General Hospital; The University of Texas at Austin; Massachusetts General Hospital

**Keywords:** working alliance, depression, older adults, peer intervention, clinical

## Abstract

**Objective:**

Peer support is an evidence-based intervention for depression, yet the mechanisms underlying its effectiveness remain understudied, particularly in randomized clinical trials. This study examined the mediating role of working alliance components (task, bond, and goal) for 45 participants who were administered the Peer Enhanced Depression Care (PEERS) intervention.

**Methods:**

These participants received eight weekly peer support meetings focused on enhancing self-care and increasing coping skills. Working alliance was assessed by both participants and peer coaches using the Working Alliance Inventory on a weekly basis during the 8-week intervention period. Structural equation modeling was used to assess the trajectory of working alliance subscales and whether the subscales (bond, agreement on tasks and goals) mediated depressive symptoms (PHQ-9 score).

**Results:**

Descriptive analysis showed that participants’ and peer coaches’ perceptions of working alliance differed on all three subscales (bond, agreement on goals and tasks) initially and increased and converged throughout the intervention period. Only agreement on goals as rated by peer coaches significantly mediated the relationship between the intervention and depressive symptoms. No participant-rated alliance subscales were significant mediators.

**Conclusions:**

Findings suggest that although bond may be a critical factor in alleviating symptoms of depression, agreement on goals may be a more crucial driver of change than emotional connection or agreement on tasks. Implications for intervention design and peer training include the importance of content and training on collaborative goal-setting in addition to relationship building with the participant to maximize clinical effectiveness.

**Trial Registration:**

This study was registered through ClinicalTrials.gov under registration number 2022P001675 under grant R01MH123165 on February 17th, 2020.

## Introduction

Peer support is evidence-based, widely practiced, and supported by policy. Meta-analyses have been conducted across *randomized controlled trials* in mental health demonstrating positive outcomes [1]. Peer support is offered across a range of settings from community mental health clinics to inpatient hospital settings [2] and policy supports peer support services, including Medicaid reimbursement [3]. Although the evidence of effectiveness in peer support is strong and practice is widespread, the mechanism of peer support is unclear. There is growing qualitative and quantitative evidence that working alliance, a core relational factor often studied in psychotherapy also arises in peer support. Working alliance refers to the collaborative relationship between helper and client and is comprised of three interrelated components: agreement on goals, consensus on tasks, and development of an affective bond [1–3]. A positive working alliance provides alignment between client and therapist, so that the stronger the alliance scores are, the more effective the outcomes are predicted to be [4].

Traditionally working alliance has been applied in therapist–patient relationships [5], however, the concept has been applied across disciplines. Studies in psychotherapy have found that stronger working alliance predicts positive outcomes in reducing depression and anxiety [6–9]. Working alliance is often studied from perspectives of both the client and therapist, with studies showing that convergence of patient and therapist ratings can occur. Working alliance has also been applied in areas such as case management, where positive working alliance have been found to be associated with outcomes such as quality of life, symptom severity, and attitudes toward medications in randomized controlled trials [4, 10–13].

Working alliance in peer support interventions may function differently in traditional therapist–client relationships, compared to peer interventions that draw upon shared lived experiences rather than formal clinical training [14–18]. Shared experience can decrease stigma, increase self-disclosure, and potentially increase activation for improving depression self-care behaviors [19–21]. Identification of peer and client based on shared experience may quickly enable trust and bonding to be established, but there may also be challenges in maintaining appropriate role boundaries and the effect of the shared lived experience on agreement on goals has not been well studied [22].

The effectiveness of peer interventions is thought to depend on the strength of the relationship between peer supporters and participants [23]. The importance of bonding in peer support has not been studied, although is often posited as what is most important in peer-client relationships; however, empathy and sharing experiences alone may not impact health outcomes such as depression. Factors such as mutual accountability [24], degree of collaboration and mutual understanding [25, 26], may be a key factor in sustaining peer relationships. Alignment of goals and tasks may be critical for translating supportive interactions into positive mental health outcomes. While some researchers have examined the function of working alliance in peer support contexts [16, 27], further research is needed to understand the underpinnings that guide these relationships and their application in peer support interventions.

The goal of this study was to examine whether working alliance components mediate the effect of a peer intervention on depressive symptoms among older adults. We focused on working alliance as a potential mechanism of change in a peer support intervention for older adults with depression in a randomized controlled trial. We hypothesized that stronger bonds within the working alliance would be associated with lower depressive symptom scores across the intervention. Assessing mediation effects can clarify the extent to which engagement, goal alignment, and/or emotional connection contribute to improvements in depressive symptoms and the findings may also inform the design and implementation of training programs aimed at improving peer intervention outcomes.

## Methods

### Study Design

We used the data collected from participants in the intervention arm of a two-arm parallel randomized controlled trial in which we tested a peer support intervention called PEERS, which consisted of 8 weekly 45–60 minutes telephone meetings with a peer coach [28] aimed at decreasing depression among older adults. Meetings focused on provision of social support [emotional, appraisal, and informational), goal setting and self-care, improving coping skills and information on community-based services [29]. Peer coaches completed 20 hours of training and participated in weekly group supervision sessions throughout the intervention period. Peer coaches were predominantly female and ranged in age from 51 to 70 years. Most identified as African American, had college or graduate education, and were actively engaged in employment or volunteer work. Fidelity assessments were implemented throughout the intervention to ensure its validity [29].

### Participants

Participants who received the Peers intervention were included in this study. Older adults were eligible if they were aged 50+, reported depressive symptoms (the Patient Health Questionnaire [PHQ-9] ≥ 5) (30), were cognitively intact, were persons of color or had an annual income less than 200% of federal poverty level (<$24,120), spoke English, and were able to give informed consent. Older adults with suicidal ideation, psychosis, substance use disorder, a change in psychiatric medication in the past 3 months or who were actively receiving psychotherapy more than once a month in the past 3 months were excluded. The study was approved by the Institutional Review Board of Johns Hopkins University and registered in ClinicalTrials.gov (NCT04319094).

### Assessments

Socio-demographic characteristics such as gender, age, and education were obtained. Participants were assessed for their depressive symptoms at baseline, post-intervention (8 weeks), 3, 6, 9, and 12 months. Depressive symptoms were measured with the PHQ-9 [30], which is well validated and used in clinical and research settings. PHQ-9 scores range from 0–27, with 0–5 (minimal), 6–10 (mild), 11–15 (moderate), 16–20 (moderately severe), and > 20 (severe) [30]. The PHQ-9 has demonstrated strong psychometric properties in prior research, with internal consistency estimates typically ranging from α = .86 to .89 [30]. Within our study we found good reliability (Cronbach’s α = .774).

The Working Alliance Inventory (WAI) is a validated measure of therapeutic alliance that assesses the quality of the collaborative relationship between peer coach and participant [31]. It includes three subscales which evaluate agreement on intervention tasks, the emotional bond, and agreement on goals. WAI consists of 12 items each rated on a 7-point Likert scale ranging from never (1) to always (7), with higher scores indicating a stronger alliance. Both peer coaches and participants completed the WAI weekly during the 8-week intervention. For each participant-week observation, subscale scores were computed as the mean of their respective items: task, bond, and goal. The Working Alliance Inventory (WAI) has demonstrated strong internal consistency, with Cronbach’s alpha estimates of .93 for the client version and .87 for the counselor version, and subscale alphas ranging from .85 to .88 for clients and .68 to .87 for counselors [31]. In our study, reliability was also strong, with Cronbach’s alpha values of .904 for Participant WAI and .797 for Peer WAI.

### Data Analysis

We used structural equation modeling (SEM) to examine whether the effect of the PEERS intervention on depressive symptoms (measured by PHQ-9) was mediated by the components of the working alliance, as rated separately by peer coaches and participants at baseline (Week 1) and post-intervention (Week 8). Analyses used task, bond, and goal subscales from the weekly Working Alliance Inventory (WAI) ratings. For each week in which the WAI was completed, subscale scores were calculated by averaging the items corresponding to each subscale (Task = items 1, 2, 8, 12; Bond = items 3, 5, 9, 11; Goal = items 4, 6, 7, 10). If a participant was missing Week 1 or Week 8, the closest available week was substituted (e.g., Week 2 for missing Week 1; Week 7 for missing Week 8). Descriptive analyses were conducted to summarize participant demographics, baseline depressive symptoms, and working alliance ratings. Weekly WAI ratings for task, bond, and goal subscales were included as simultaneous mediators in a multivariate SEM model, controlling for baseline demographic characteristics (age, gender, education, marital status, race, and ethnicity). Figure 2 illustrates the analytical mediation model, showing the effects of three components of the working alliance (task, bond, and goal), including a possible direct effect of intervention on depression symptoms. Since depressive symptoms were measured before and after the intervention, the total effect of ‘time’ in the model represents the impact of the intervention. Unstandardized partial regression coefficients (β) are displayed along each path. In this longitudinal SEM model, each regression coefficient associated with “Time” is effectively the difference between the mean of the pertinent dependent variable at Week 8 and for baseline/Week 1 (reference) timepoint, adjusted for other covariates in the model. Indirect effect estimates are essentially the product of multiple regression coefficients associated with each arrow along the indirect route.

Analyses were conducted in R using the lavaan package, with cluster-robust standard errors to account for repeated measures within participants. For variables in which their mediated (indirect) effect on depression symptoms was in the same direction as the total effect of the intervention, the proportion of the total effect mediated by the given variable was calculated by dividing indirect effect by total effect. Missing data were assessed for each WAI subscale (goal, bond, and task) across 344 entries per subscale. The percentage of missing data was 3.2% for goal, 5.8% for bond, and 10.8% for task. Together, WAI had an overall missing rate of 6.6%. Structural equation models were estimated using complete-case analysis by default, meaning only cases with complete data on all model variables were included in the estimation.

## Results

Table 1 shows that the mean age of the participants was 70 (SD 9.59). The majority (86.7%) were women, not married (82.2%), and lived alone (64.4%), and more than half were non-White. Educational levels were evenly distributed among high school, college, and graduate degrees. Depression symptoms were in the low-moderate range (11.5, SD 5.00).

**Figure 1:**
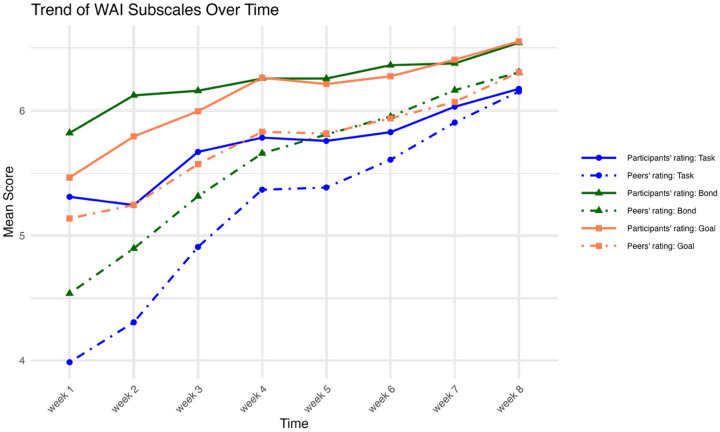
Means for Working Alliance Subscales over Time for the Intervention Condition, for Participants and PEERS.

Figure 1 presents weekly trends in mean working alliance subscale scores as reported by both peer coaches and participants. Overall, ratings increased steadily across all subscales (task, bond, and goal) over the eight-week intervention period. Participant-reported alliance scores were consistently higher than those reported by peer coaches. Ratings from both participants and peer coaches showed converging and upward trajectories, suggesting a strengthening of the therapeutic alliance over time.

**Figure 2:**
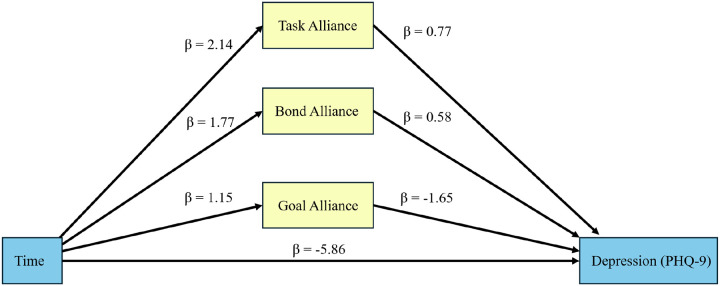
Structural Equation Model Representation of Peer Working Alliance Inventory Mediation Effects. (b = unstandardized partial regression coefficient).

For peer coach-rated alliance scores, the time/intervention led to a significant reduction in depressive symptoms (β for the total effect = −5.07, 95% CI: (−6.506, −3.631), *p* < 0.001), with a large and significant direct effect (β = −5.86, 95% CI: (−7.703, −4.025), *p* < 0.001). Among the three indirect pathways, only agreement on goals showed a statistically significant indirect effect (estimate = − 1.90, 95% CI: (−3.707, −0.087), *p* = 0.040), indicating improvement in goal agreement between peer coaches and participants partially accounted for the intervention’s total impact on reducing depression. The mediating effect of agreement on goals augmented the direct effect of time/intervention symptoms, resulting in a greater decrease in depression across time than would have been due to the effect of the time/intervention alone. The percent of the total effect mediated via the agreement-on-goals alliance was 37.4%. The task and bond subscales had non-significant indirect effects (estimates = 1.66, 95% CI: (−1.711, 5.026) and estimates = 1.04, 95% CI: (−1.999, 4.068), respectively). Direct effect of the intervention appears stronger (in absolute value) than the total effect because conflicting positive indirect effects of task and bond alliance are diluting the total negative effect. The path diagram for the structural equation model with peer-rated alliance scores as mediators is presented in Fig. 2. Note that the effect of Time on each WAI subscale was positive (i.e., mean subscale values improved across time), but only the goal alliance subscale had a further adjusted negative impact on depression (PHQ-9).

In contrast to the peer-coach rated alliance subscales, none of the participant-rated alliance subscales showed statistically significant mediation (Table 2). As with the peer coach ratings, the direction and magnitude of some indirect paths differed from the direction of the total effect. Note that the indirect effect for the participant-rated goal alliance subscale approached marginal statistical significance with a counterintuitive positive estimate (estimate = 2.23, 95% CI: (−0.196, 4.663), *p* = 0.072).

## Discussion

This study examined whether the working alliance mediated the impact of a peer-delivered psychosocial intervention for older adults with depression symptoms. Our hypothesis was not supported as the bond rating from the peer coach and participant was not a significant mediator. We found that goal alignment, specifically peer coach-rated agreement on goals, was the only factor that mediated changes in depression symptoms over time. In instances where peer coaches rated goal alignment more strongly, participants reported lower depression symptoms over time. Participant-rated alliance subscale scores and peer coach subscales for task or bond did not show a similar effect. The finding highlights goal alignment as a mechanism of change, challenging traditional expectations that prioritize relational bonds as the primary driver of improvement for peer support interventions [32]. These findings suggest that in the context of peer support, a shared understanding of goals from a trained peer coach may be more important for clinical outcomes than emotional connection alone or even the participant’s perception of goal alignment.

Although bonds are often viewed as central to a working alliance [2], these findings suggest that peer coaches, perhaps due to their prior experience and study training, had a clearer sense of progress than participants. These results are similar to previous research in psychotherapy that shows incongruence between therapist and client ratings of working alliance [33–34]. Further support of this divergence can be seen from scores across time (seen in Fig. 1). At baseline, working alliance rating scales differed between participant and peer coaches, with peer coaches rating the WAI lower than study participants. However, as the intervention progressed, their WAI scores converged, which may indicate the different expectations and understanding of their work together initially. This aligns with previous research indicating that working alliance scores are rated differently at the beginning of an intervention but converge over its course [9].

Previous research on peer support has often emphasized empathy, shared experience, and emotional connection as mechanisms for change [35–36]. While these elements are clearly important for building trust and engagement, findings suggest that they may not be sufficient to produce symptom improvement on their own. Instead, it appears when peer coaches believe they are aligned with participants on goals, clinical improvements are more likely. These results are consistent with models of behavior change that emphasize the importance of goal setting and action planning [37]. Future studies could test strategies for strengthening agreement on goals between peer coaches and participants to determine how these approaches influence symptom improvement.

Although bond and task alliance both improved over time, these components did not emerge as significant mediators of change in relation to depressive symptoms among older adults. While participants and peer coaches became more aligned on bonds and tasks throughout the intervention, these alliance components did not contribute to the relationship with decreased depressive symptoms when the intervention ended. One possible explanation might be a product of the peer intervention relationship itself. Peer coaches differ from clinicians insofar as their support is based primarily on shared lived experience rather than formal training or professional authority [14–15]. As a result, while participants may perceive the peer relationship as beneficial for social connection [38], it may lack the influence that a therapist might exert in a clinical setting. In this context, emotional bonding may help foster trust and engagement [39] but does not necessarily translate into measurable changes in outcomes. Clarity, specificity, and mutual agreement around goals from the peer coach perspective are more directly linked to symptom reduction. This points to the possibility that in peer-delivered interventions, the most effective component appears to be the extent to which peer coaches perceive collaborative alignment on goals, rather than emotional connection.

### Strengths and Limitations

This study has several strengths. It utilized psychometrically validated measures of working alliance [31] and depressive symptoms [30] and implemented a fidelity-monitored peer support intervention [40], ensuring methodological rigor. Alliance ratings from both peer coaches and participants provided a detailed view of alliance trajectories and their potential mediating role in symptom change. The analytic approach using structural equation modeling with cluster-robust standard errors enhanced precision and accounted for repeated measures. The sample was drawn from a specific population of socioeconomically disadvantaged older adults, which is both a strength but also limits generalizability to other groups. In terms of limitations, a primary limitation was the small sample size given mediation analyses, which reduces statistical power and may affect stability of estimates. Future research should consider using correction methods. Additionally, the lack of a control group for this study means that observed effects could be confounded by changes over time due to other factors rather than the intervention itself. Future research should replicate these findings with larger samples to improve sensitivity and external validity.

## Conclusion and Implications

This study offers important insights into the mechanisms driving clinical improvement in peer support interventions for older adults with symptoms of depression. While emotional connection and shared experience have traditionally been viewed as central to peer support, findings suggest that peer coach perceptions on goal alignment are a more critical factor in symptom reduction. This challenges conventional thinking that prioritizes relational bonds [32] and underscores the importance of structuring peer interventions around clear, collaborative goals. Specifically, training peer coaches to identify, communicate, and reinforce shared goals may amplify the therapeutic impact of peer support interventions.

## Figures and Tables

**Table 1:** Clinical, Demographic, and Alliance Characteristics Table (N = 45)

Variable	N (%) / Mean (SD)
**Age**	
Mean (SD)	70.0 (9.59)
Median [Min, Max]	68.0 [54.0, 95.0]
**Gender**	
Female	39 (86.7%)
Male	6 (13.3%)
**Education Level**	
High school or lower	18 (40.0%)
College	20 (44.4%)
Postgraduate	7 (15.6%)
**Marital Status**	
Married	8 (17.8%)
Not Married	37 (82.2%)
**Race**	
White	19 (42.2%)
Black	21 (46.7%)
Mixed	4 (8.9%)
Other	1 (2.2%)
**Ethnicity**	
Non-Hispanic	41 (91.1%)
Hispanic	3 (6.7%)
Missing	1 (2.2%)
**Household Status**	
Lives Alone	29 (64.4%)
With Others	16 (35.6%)
**Counseling History**	
No	13 (28.9%)
Yes	32 (71.1%)
**PHQ-9**	11.5 (5.00)
**Participant Working Alliance**	
Goal	5.46 (1.32)
Task	5.31 (1.10)
Bond	5.82 (1.29)
**Peer Coach Working Alliance**	
Goal	5.14 (0.948)
Task	3.98 (1.33)
Bond	4.53 (1.30)

**Table 2:** Mediation Model: Peer and Participant-Rated Alliance Subscales as Simultaneous Mediators of Intervention Effects on Depressive Symptoms

Peers Rating	Effect Estimate	SE	95% CI	p-value
Indirect Effect: WAI_Task	1.657	1.719	(−1.711, 5.026)	0.335
Indirect Effect: WAI_Bond	1.035	1.548	(−1.999, 4.068)	0.504
Indirect Effect: WAI_Goal	−1.897	0.924	(−3.707, −0.087)	0.040
Total Indirect Effect	0.795	0.886	(−0.942, 2.532)	0.369
Direct Effect	−5.864	0.938	(−7.703, −4.025)	< .001
Total Effect	−5.069	0.733	(−6.506, −3.631)	< .001
**Participant’s Rating**				
Indirect Effect: WAI_Task	−0.412	0.521	(−1.434, 0.61)	0.429
Indirect Effect: WAI_Bond	−1.410	0.982	(−3.335, 0.515)	0.151
Indirect Effect: WAI_Goal	2.234	1.240	(−0.196, 4.663)	0.072
Total Indirect Effect	0.412	0.589	(−0.742, 1.566)	0.484
Direct Effect	−5.786	0.965	(−7.677, −3.895)	< .001
Total Effect	−5.374	0.769	(−6.881, −3.866)	< .001

Note: WAI = Working Alliance Inventory

## Data Availability

Data is available at the NIMH Data Archive.

## References

[1] Smit D, Miguel C, Vrijsen JN, : The effectiveness of peer support for individuals with mental illness: systematic review and meta-analysis. Psychol Med 2023; 53:5332–5341 36066104 10.1017/S0033291722002422 PMC10476060

[2] Joo JH, Bone L, Forte J, : The benefits and challenges of established peer support programmes for patients, informal caregivers, and healthcare providers. Family Practice 2022; 39:903–912 35104847 10.1093/fampra/cmac004 PMC9508871

[3] Medicaid Behavoral Health Services: Peer Support Services, Kaiser Family Foundation, 2022

[4] Solomon P, Draine J, Delaney MA: The working alliance and consumer case management. The journal of mental health administration 1995; 22:126–134 10142126 10.1007/BF02518753

[5] Horvath AO, Wiseman H, & Tishby O: The psychotherapy relationship: Where does the alliance fit? In Developing the Therapeutic Relationship (pp. 15–28). American Psychological Association 2018; 10.1037/0000093-002

[6] Wu MS, Wickham RE, Chen SY, : A large-scale evaluation of therapeutic alliance and symptom trajectories of depression and anxiety in blended care therapy. PLoS ONE [Internet]. 2024 Nov 8;19(11):e0313112. 39514467 10.1371/journal.pone.0313112 PMC11548720

[7] Kondrat DC, Early TJ: An exploration of the working alliance in mental health case management. Social Work Research [Internet]. 2010 Dec 1;34(4):201–211.

[8] Arnow BA, Steidtmann D, Blasey C, : The relationship between the therapeutic alliance and treatment outcome in two distinct psychotherapies for chronic depression. Journal of Consulting and Clinical Psychology [Internet]. 2013 Jan 1;81(4):627–638. 23339536 10.1037/a0031530 PMC3742444

[9] Laws HB, Constantino MJ, Sayer AG, : Convergence in patient–therapist therapeutic alliance ratings and its relation to outcome in chronic depression treatment. Psychotherapy Research [Internet]. 2016 Feb 1;27(4):410–424. 26829714 10.1080/10503307.2015.1114687 PMC4969229

[10] De Leeuw M, Van Meijel B, Grypdonck M, : The quality of the working alliance between chronic psychiatric patients and their case managers: process and outcomes. Journal of psychiatric and mental health nursing 2012; 19:1–7 22070798 10.1111/j.1365-2850.2011.01741.x

[11] Howgego IM, Yellowlees P, Owen C, : The therapeutic alliance: The key to effective patient outcome? A descriptive review of the evidence in community mental health case management. Australian & New Zealand Journal of Psychiatry 2003; 37:169–183 12656956 10.1046/j.1440-1614.2003.01131.x

[12] Roebuck M, Latimer E, Bergeron-Leclerc C, : The Working Alliance as a Mediator Between Fidelity to Strengths Model Case Management and Client Outcomes. Psychiatr Serv 2022; 73:1248–1254 35502516 10.1176/appi.ps.202100387

[13] Nath SB, Alexander LB, Solomon PL: Case managers’ perspectives on the therapeutic alliance: a qualitative study. Social Psychiatry and Psychiatric Epidemiology [Internet]. 2012 Feb 16;47(11):1815–1826. 22349149 10.1007/s00127-012-0483-z

[14] Fortuna KL, Solomon P, Rivera J: An update of Peer Support/Peer provided services underlying processes, benefits, and critical ingredients. Psychiatric Quarterly [Internet]. 2022 Feb 18;93(2):571–586. 35179660 10.1007/s11126-022-09971-w PMC8855026

[15] Solomon P: Peer Support/Peer provided services underlying processes, benefits, and critical ingredients. Psychiatric Rehabilitation Journal [Internet]. 2004 Jan 1;27(4):392–401. 15222150 10.2975/27.2004.392.401

[16] Mead S, Hilton D, Curtis L: Peer support: A theoretical perspective. Psychiatric Rehabilitation Journal [Internet]. 2001 Jan 1;25(2):134–141. 11769979 10.1037/h0095032

[17] Stefancic A, House S, Bochicchio L, : “What we have in Common”: A qualitative analysis of shared experience in Peer-Delivered services. Community Mental Health Journal [Internet]. 2019 Mar 22;55(6):907–915. 30903534 10.1007/s10597-019-00391-y

[18] Tang JPS, Liu T, Lu S, : ‘It was the deepest level of companionship’: peer-to-peer experience of supporting community-dwelling older people with depression - a qualitative study. BMC Geriatrics [Internet]. 2022 May 19;22(1):443. 35590279 10.1186/s12877-022-03121-4 PMC9121547

[19] Truong C, Gallo J, Roter D, : The role of self-disclosure by peer mentors: Using personal narratives in depression care. Patient Educ Couns 2019; 102:1273–1279 30791990 10.1016/j.pec.2019.02.006 PMC6546521

[20] Sun J, Yin X, Li C, : Stigma and peer-led interventions: a systematic review and meta-analysis. Frontiers in Psychiatry 2022; 13:915617 35865307 10.3389/fpsyt.2022.915617 PMC9294224

[21] Joo JH, Xie A, Choi N, : Loneliness, Self-Efficacy and Adaptive Coping: Mixed Methods Analysis of Mediation in a Peer Support Intervention for Depression. Am J Geriatr Psychiatry 2025; 10.1016/j.jagp.2025.02.013 PMC12085289 40121127

[22] Knopes J, Dégale-Flanagan M: Boundary Flexibilities in Mental Health Peer Support: The Peer Perspective. Journal of Psychosocial Rehabilitation and Mental Health 2023; 10.1007/s40737-023-00379-8

[23] Mikolajczak-Degrauwe K, Slimmen SR, Gillissen D, : Strengths, weaknesses, opportunities and threats of peer support among disadvantaged groups: A rapid scoping review. International Journal of Nursing Sciences [Internet]. 2023 Sep 15;10(4):587–601. 38020843 10.1016/j.ijnss.2023.09.002 PMC10667317

[24] Fortuna KL, Brooks JM, Umucu E, : Peer Support: a Human Factor to Enhance Engagement in Digital Health Behavior Change Interventions. Journal of Technology in Behavioral Science [Internet]. 2019 May 29;4(2):152–161. 34337145 10.1007/s41347-019-00105-x PMC8323847

[25] Martin DJ, Garske JP, Davis MK: Relation of the therapeutic alliance with outcome and other variables: a meta-analytic review. J Consult Clin Psychol. 2000 Jun;68(3):438–50. 10883561

[26] Wampold BE: How important are the common factors in psychotherapy? An update. World Psychiatry [Internet]. 2015 Sep 25;14(3):270–277. 26407772 10.1002/wps.20238 PMC4592639

[27] Joo JH, Hwang S, Gallo JJ, : The impact of peer mentor communication with older adults on depressive symptoms and working alliance: A pilot study. Patient Education and Counseling [Internet]. 2017 Oct 23;101(4):665–671. 29128295 10.1016/j.pec.2017.10.012 PMC8969792

[28] Joo JH, Hwang S, Abu H, : An Innovative Model of Depression Care Delivery: Peer Mentors in Collaboration with a Mental Health Professional to Relieve Depression in Older Adults. American Journal of Geriatric Psychiatry [Internet]. 2016 Feb 10;24(5):407–416. 10.1016/j.jagp.2016.02.002 PMC5116434 27066731

[29] Joo JH, Davey-Rothwell M, Choi N, : Increasing the repertoire for depression care: Methods and challenges of a randomized controlled trial of peer support for vulnerable older adults. American Journal of Geriatric Psychiatry [Internet]. 2023 Feb 2;31(8):586–595. 10.1016/j.jagp.2023.01.023 PMC10329981 36842891

[30] Kroenke K, Spitzer RL, Williams JBW: The PHQ-9: validity of a brief depression severity measure. Journal of General Internal Medicine [Internet]. 2001 Sep 1;16(9):606–613. 11556941 10.1046/j.1525-1497.2001.016009606.x PMC1495268

[31] Horvath AO, Greenberg LS: Development and validation of the Working Alliance Inventory. Journal of Counseling Psychology [Internet]. 1989 Apr 1;36(2):223–233.

[32] Pérez-Rojas AE, González JM, Fuertes JN: The bond of the Working Alliance. Oxford University Press eBooks [Internet]. 2019. p. 11–42.

[33] Bachelor A: Clients' and therapists' views of the therapeutic alliance: similarities, differences and relationship to therapy outcome. Clin Psychol Psychother. 2013 Mar-Apr;20(2):118–35. doi: 10.1002/cpp.792. Epub 2011 Nov 14. 22081490

[34] Reis BF, Brown LG: Reducing psychotherapy dropouts: Maximizing perspective convergence in the psychotherapy dyad. Psychotherapy [Internet]. 1999 Jan 1;36(2):123–136.

[35] Kirkegaard S, Andersen D: Peer workers as emotion managers: Tight and loose enactment of mutuality in mental health care. SSM - Qualitative Research in Health [Internet]. 2022 Nov 25;2:100200.

[36] Shalaby R a H, Agyapong VIO: Peer Support in Mental Health: Literature review. JMIR Mental Health [Internet]. 2020 Feb 15;7(6):e15572 32357127 10.2196/15572 PMC7312261

[37] Bailey RR: Goal setting and action planning for health behavior change. American Journal of Lifestyle Medicine [Internet]. 2017 Sep 13;13(6):615–618. 31662729 10.1177/1559827617729634 PMC6796229

[38] Schwei RJ, Amesoudji AW, DeYoung K, : Older adults' perspectives regarding peer-to-peer support programs and maintaining independence. Home Health Care Serv Q. 2020 Oct-Dec;39(4):197–209. doi: 10.1080/01621424.2020.1778594. Epub 2020 Jun 11. 32525461 PMC8227957

[39] Stubbe DE: The therapeutic alliance: the fundamental element of psychotherapy. FOCUS the Journal of Lifelong Learning in Psychiatry [Internet]. 2018 Oct 1;16(4):402–403. 10.1176/appi.focus.20180022 PMC6493237 31975934

[40] Solomon P, Draine J, Delaney MA: The use of restraining orders by families of severely mentally ill adults. Administration and Policy in Mental Health and Mental Health Services Research [Internet]. 1995 Nov 1;23(2):157–161.

